# Updates in the pathophysiology of COVID-19 infection in male reproductive and sexual health: a literature review

**DOI:** 10.3389/fendo.2023.1226858

**Published:** 2024-02-26

**Authors:** Meshari A. Alzahrani, Khalid O. Alkhani, Abdullah M. Alassaf, Jehad I. Alorainy, Saleh Binsaleh, Raed Almannie

**Affiliations:** ^1^ Department of Urology, College of Medicine, Majmaah University, Al-Majmaah, Saudi Arabia; ^2^ College of Medicine, King Saud University, Riyadh, Saudi Arabia; ^3^ Department of Surgery, Urology Division, College of Medicine, King Saud University, Riyadh, Saudi Arabia

**Keywords:** men, COVID-19, SAR-CoV-2, sexual health, reproductive health, pathophysiology

## Abstract

This extensive comprehensive review explores the impact of the Coronavirus disease 2019 (COVID-19) pandemic on men’s sexual and reproductive health. We conducted a literature review focusing on the possible pathophysiology by which severe acute respiratory syndrome corona virus 2 (SARS-CoV-2) affects men’s sexual and reproductive systems. We reviewed most of the studies that reported the impact of SARS-CoV-2 infection on the Testicular, Epididymal, Prostatic, and Penile tissue. Also, we focused on evaluating the SARS-CoV-2 infection on semen parameters and male reproductive hormones. Finally, we reviewed the COVID-19 vaccine’s effect on male reproductive and sexual health. Findings revealed the adverse consequences of SARS-CoV-2 at cellular and organ levels on the male genital tract. However, the reported data are still controversial. The initial data regarding COVID-19 vaccination was promising promoted safety for men’s reproductive and sexual health. We conclude this paper by offering recommendations to address these adverse consequences and potentially improve sexual and reproductive health among men in the post-COVID-19 pandemic era.

## 1 Introduction

Coronavirus disease 2019 (COVID-19), first detected in China in December 2019, has since spread globally, with 132 million reported cases and 3 million deaths as of April 2021. (1, 2). The first publications from China and Italy reported higher fatality rates in men than in women due to the pandemic (3). New data reported in November 2021 continue to show that men have a more significant share of hospitalizations (55%), Intensive Care Unit (ICU) admissions (63%), and deaths (57%) (4).

SARS-CoV-2 infects cells through its spike protein binding to angiotensin-converting enzyme 2 (ACE2), with host transmembrane protease serine 2 (TMPRSS2) playing a crucial role in activating and cleaving the S protein as the virus binds to ACE2 (5). ACE2 is expressed in many organs and tissues. Studies have found that ACE2 and TMPRSS2 are expressed abundantly in testis somatic cells, spermatogonia, and peritubular myoid cells (6). Thus, a high level of susceptibility to SARS-CoV-2 for the testis has been established. However, there is controversy surrounding this conclusion. According to other studies ACE2 does not co-express with TMPRSS2 in testicular cells, supporting the notion that testicular cells are not highly susceptible to viral infection (7–9). SARS-CoV-2 entry and priming can be affected by testosterone and are linked to a weaker immune response, higher infection rates, and thromboembolic predisposition in male hosts (10). Compared with female subjects, Males appear to have a slower clearance rate of SARS-CoV-2, possibly as a result of higher ACE2 levels in the testis (11).

These findings have the potential to be very important for male sexual health; indeed, based on this preliminary evidence, there is quite enough evidence to hypothesize that the consequences of COVID-19 can extend to sexual and reproductive health. A retrospective study involving 1099 cases showed that the percentage of male COVID-19 patients was nearly 60%, and around 55% of them were reproductive-aged (15-49 years old). Therefore, concern was raised about whether SARS-CoV-2 may affect the male reproductive system (12, 13).

In this review, we aim to summarize the latest scientific updates on the effects of the novel coronavirus on several aspects of male reproductive health and fertility and to discuss the theories explaining the pathophysiology of these effects.

## 2 Effect of SARS-CoV-2 on reproductive tissues

### 2.1 Testicular tissue

The pathological effects of SARS-CoV-2 infection on testicular tissue have been studied through molecular tests on deceased COVID-19 patients. The most common are Reverse transcription polymerase chain reaction (RT-PCR), Immunohistochemistry (IHC), and Transmission electron microscopy (TEM). Bian et al. (14) reported during the earlier stages of the pandemic that in deceased patients, SARS-CoV-2 was detected through RT-PCR. Since that study, conflicting evidence on the presence of SARS-CoV-2 in testicular histology has been published. Despite another study also confirming positive SARS-CoV-2 RT-PCR in the testis, three more recent studies reported a negative test in either all or the majority of their subjects (15–18). Masterson et al. (18) hypothesize that their adverse finding conflicted with previous studies due to the method of harvesting testicular tissue. At the same time, they used an open fashion in contrast to previous studies, which used a percutaneous biopsy which may raise concerns for potential contamination. Bian et al. (14) also reported positive IHC staining. However, IHC in the context of SARS-CoV-2 positive participants remains ambiguous. This is because there is no positive control for IHC staining, studies that reported positive findings have not shown a significant correlation within positive SARS-CoV-2 RT-PCR participants, and the molecules targeted in IHC staining have not been consistent throughout all the previous studies (15–18). Despite these higher levels of ACE2, IHC staining has been correlated with worse clinical outcomes regarding testis injury severity (19).

Similarly, reported data on the histopathological effect of SARS-CoV-2 on the testis have been conflicting. While these differences could be secondary to different methodologies in conducting the biopsies and reporting them, other theories should be considered. Bian et al. (14) initially said various degrees of injury to the testis and reduced spermatogenesis. Shortly after that study, another publication reported negative histopathological findings, which the authors attributed to the duration after the infection of which they obtained the biopsy. They hypothesize that the injury to the testis might have occurred at the earlier stage of the disease and have resolved later (15). Other studies with a shorter duration between the onset of symptoms and biopsy showed varying degrees of injury, including interstitial edema, vascular changes, and germ cell loss (16, 17). Finally, Masterson et al. (18) found no injury to testicular tissue in postmortem biopsies of deceased patients following SARS-CoV-2 infection. Their study was the first to conduct the biopsy using an open fashion, contrary to a percutaneous biopsy performed by a previous study. The authors hypothesize that positive findings reported previously could be secondary to contamination during a percutaneous biopsy. Summary for reported studies about impact of SARS-CoV-2 infection on testicular function at (**Table 1**).

**Table 1:** Testicular histological features of patient diagnosed with COVID-19.

Author/year	No. of COVID -19 subjects	Testicular biopsy technique	Timing of sampling	Number of cases tested positive RT-PCR in testis	Reported COVID-19 IHC	EM/TEM	Confirmed testicular injury
Bian et al., 2020 (14)	91	Percutaneous biopsy	Not reported	Not reported	+ ACE2	Positive staining	Detected injury
Duarte-Neto et al., 2022 (17)	11	Percutaneous biopsy	Not reported	3	Sars-Cov-2 N-protein	Positive staining	Detected Injury
Ma et al., 2021b (16)	5	Not reported	Not reported	2	Not reported	Positive staining	Not reported
Masterson et al., 2022 (18)	8	Open biopsy	Not reported	1	Equivocal	Not reported	No inflammation
Yang et al., 2020 (15)	12	Incisional/Percutaneous biopsy	Within 1 hours	1	+ CD3, + CD68,+ ACE2	Not detected	Detected Injury
Achua et al., 2021 (19)	6	Not reported	Within 24-48 hours	Not reported	Not reported	Positive staining in 2 cases	Presence of lymphocytes and macrophages

RT-PCR; Reverse transcription polymerase chain reaction, IHC; immunohistochemistry, EM/TEM; Transmission electron microscopy (TEM).

### 2.2 Epididymal tissue

The limited expression of ACE2 in the epididymis has been reported (20). However, highly expressed receptors such as Neuropilin 1 (NRP1) and Cluster of Differentiation 147 (CD147) in the epididymis has been reported (21). Evidence suggest that the expression of the mentioned receptors play a significant role in the entry of SARS CoV 2 to host cells (22, 23). In addition, SARS-CoV-2’s spike protein can bind to epididymal sperm (24). Analysis of the epididymis in deceased COVID-19 patients found many immature spermatocytes and sperm accumulated in the cauda (25). Orchiepididymitis was diagnosed in a pediatric patient with COVID-19 who presented with testis swelling and epididymal inflammation with reactive hydrocele (26). Similarly, La Marca et al. (27) reported COVID-19-induced epididymitis symptoms, such as slight swelling and vascularization accentuation in the epididymis. COVID-19-induced epididymitis presents as a reactional hydrocele with nonuniform echo or microcyst dissemination, resulting in caput augmentation (>1.2 cm) and scrotum incrassation (28).

### 2.3 Prostatic tissue

Prostatic fluid, also known as expressed prostatic secretion (EPS), is a vital component of semen secreted by the prostate gland, and it accounts for around 33% of the volume of ejaculation. Since ACE2 and TMPRSS2 are highly expressed in the epithelium of the human prostate, it is plausible to assume that SARS-CoV-2 could impact the prostate (29). The Androgen Receptor (AR) is vital in managing cellular activities associated with prostate function and physiology. Prostate cancer is directly connected to imbalances in androgen and AR signaling (30). Moreover, new studies suggest that ACE2 could be controlled by AR signaling (31, 32). Behavioral factors like smoking and obesity, as well as comorbidities such as diabetes, hypertension, and alcoholism, are known to affect COVID-19 severity as well as the progression and outcomes of prostate cancer (33–37). The most prominent shared risk factor for prostate cancer and COVID-19 complications and mortality is age, with men over 50 being at higher risk for prostate cancer and more prone to severe outcomes from COVID-19 (38–41). Several studies have demonstrated that androgens can influence the range of immune responses by modifying the behavior of particular immune subsets responsible for removing viruses (42, 43). Studies involving 84 subjects found no evidence of SARS-CoV-2 RNA in the EPS (44, 45).

### 2.4 Effect on endothelial and penile tissue

Over the past few decades, a good amount of evidence supports erectile function as an excellent indicator of systemic health in general and vascular health in particular (46), effects of COVID-19 on the cardiovascular system (i.e., acute cardiac injury, myocarditis) as well growing evidence in the role of endothelial cell dysfunction during COVID-19 infection most importantly, the endothelium expresses the ACE2 led many have hypothesized there may be an increased risk of Erectile dysfunction (ED) following COVID-19 (47).

Three studies have assessed the association between COVID-19 and ED on a population level. Compared men with prior COVID-19 infection to men without documented COVID-19 infection. They all reported that newly diagnosed erectile dysfunction is higher in men with prior COVID-19 compared to age-matched control (47–49).

The pathophysiology behind this association between COVID-19 and ED has been described by a pilot study examined the histopathological features of two cases developing severe erectile dysfunction post-COVID-19 infection which revealed decreased expression of endothelial netric oxide synthase (eNOS), which is consistent with endothelial dysfunction (2). Moreover, this study reported positive spiked Coronavirus-like viral particles in the peri-vascular erectile tissue observed via TEM and Viral RNA was detected in the tissue samples using PCR (2).

## 3 Effect of SARS-CoV-2 on semen parameters and reproductive hormones

### 3.1 Semen parameters

A total of 10 observational studies that have been reviewed investigated the impact of SARS-CoV-2 infection and semen parameters (semen volume, sperm concentration, total sperm counts, percentages of total motile and progressively motile spermatozoa, percentage of normal morphology) in semen specimens collected from men who were acutely infected or those who were recovering/recovered from SARS-CoV-2 (1, 13, 45, 50–56), 9 of them reported a significant decline in one or more of the semen parameters in semen specimens of men with active or recent SARS-CoV2 infection in comparison to healthy controls or concerning WHO guidelines (57). However, the results of these studies were inconsistent regarding which semen parameter is affected, as two studies revealed a global decline in all semen parameters (50, 52), while the rest reported the change in only a few specific semen parameters that have been analyzed, Summary reported studies about the impact of COVID-19 on semen parameters are summarized in **Table 2**.

**Table 2:** COVID-19 effect on Semen parameters.

Author/year	Number of COVID-19 cases	Main conclusion
Erbay et al., 2021 (52)	69	Global Decline in all semen parameters in comparison to healthy controls
Falahieh et al., 2021 (1)	20	sperm total motility below the reference range according to WHO criteria
Gacci et al., 2021 (53)	43	total sperm counts below the reference range according to WHO criteria
Guo et al., 2021a (55)	23	all semen parameters where within normal reference range according to WHO guidelines
Guo et al., 2021b (56)	41	Decreased sperm concentration in comparison to healthy controls
Holtmann et al., 2020 (50)	34	Global Decline in all semen parameters in comparison to healthy controls
Li et al., 2020a (58)	23	Decreased sperm concentration in comparison to healthy controls
Ma et al., 2021a (13)	12	sperm total motility below the reference range according to WHO criteria
Ruan et al., 2021 (45)	74	Decreased sperm concentration, total sperm count and total motility in comparison to healthy controls
Temiz et al., 2021 (54)	30	decrease in the percentage of normal morphology in comparison to healthy controls

Guo et al. (55) revealed that all semen parameters were within normal reference range according to WHO guidelines; what distinguishes this study from the others that have been reviewed is the median interval from confirmation of SARS-CoV2 infection to providing semen samples from study subjects was only 32 days, and 52% of the subjects were still tested positive by pharyngeal swabs. This conflict between the studies suggests that SARS-CoV-2 infection is unlikely to cause semen quality to decline at the onset but rather be delayed with indirect pathophysiology. They considered the duration of human spermatogenesis, which is 78 days (55, 59).

Several theories have been put forward to explain the Impact of SARS-CoV-2 infection on semen quality; one of them is direct invasion and damage of the testicular tissue by the virus since It is known that a broad range of virus families, including human immunodeficiency virus (HIV), mumps virus, influenza, Zika virus, etc., may attack testes and affect male reproductive function (59).

Preliminary studies on SARS-CoV-2 infection had indicated the possibility of SARS-CoV-2 outreach to male gonads, suggesting the role of ACE2 as the cellular receptor for SARS-CoV-2 may be the mechanism for access to the male reproductive organs where ACE2 is predominantly (51). Therefore, theoretically regarded as a vulnerable target to SARS-CoV-2. 8 studies reported the absence of SARS-CoV-2 in all semen specimens collected from men who were acutely infected or those who were recovering/recovered from SARS-CoV-2; these reports suggest that the testis might be not a target organ for SARS-CoV-2 (13, 45, 50, 51, 54, 55, 60, 61). Contrary to these reports, two studies demonstrated SARS-Cov-2 positivity in semen samples. In contrast, one study conducted in China revealed that SARS-CoV-2 was detectable in 6 out of 38 (15.8%) semen specimens collected from male COVID-19 patients, including the patients recovered from the infection (2 out of 23, 8.7%) (58). However, this study did not describe the semen collection or analysis in detail, nor was there evidence of SARS-CoV-2 in the urine of these patients, so the possibility of viral contamination from non-semen sources could not be excluded completely; the other study conducted in Italy reported detection of one positive SARS-CoV-2 genome in semen sample after 21 days after the second negative swab (53). Li et al. (51) suggests that detecting SARS-CoV-2 in the seminal fluid is not necessarily considered an absolute determinant of the impact of SARS-CoV-2 on male fertility and semen quality. And there are multiple other probable indirect mechanisms where SARS-CoV-2 infection could affect semen quality apart from a natural condition. Carlsen et al. (62) investigated the effect of febrile illness on semen parameters during the different phases of spermatogenesis; the study found that sperm concentration, morphology, and motility were significantly affected by fever occurring during the period of meiosis and the postmitotic period (spermiogenesis), but not by fever occurring during mitotic proliferation or after completion of spermiogenesis. An effect on sperm morphology and motility can only be seen when fever occurs during spermiogenesis, where the spermatids undergo morphological changes to sperm and acquire motility. However, the study couldn’t attribute the difference due to fever or the underlying cause of febrile illness (62).

Li et al. (51) investigated levels of proinflammatory cytokines and chemokines in semen samples in recovering COVID-19 men, the study shows increased seminal levels of IL-6, TNF- a, and MCP-1 compared to control males were observed. Although the absence of RNA virus detection was demonstrated by the studies discussed earlier including the aforementioned study. Altered seminal immune markers signifying immune impairment by COVID-19 illness Li et al. (51). This suggests that impaired semen quality among COVID-19 patients could be a result of the immune response in the testis and epididymis in COVID-19 patients.

In addition to these theories, several articles have been attributed the change in semen parameters to hormonal changes (low testosterone levels) (53, 54). The impact of SARS-CoV-2 on axis male reproductive hormonal function will be discussed below in further details.

### 3.2 Reproductive hormones

Testosterone (T) and Follicle-stimulating hormone (FSH) serum levels were lower in infected males and serum Luteinizing Hormone (LH) levels were considerably higher. Also, a significant elevation in serum prolactin (PRL) levels was noted (63). It is important to point out that PRL can be influenced by many factors. Higher PRL levels may suppress the pituitary gland resulting in decreased gonadotropin levels (64). In a follow-up study of men recovering from COVID-19 over seven months, it was found that almost 90% of patients had increased total testosterone (tT) levels after recovery compared to baseline levels. However, further decreased tT levels were observed in 10% of the patients, suggesting persistent hypogonadism. Additionally, 55% of men had tT concentrations suggestive of hypogonadism, especially when comorbid conditions are present (65). Also, Apaydin et al. found that hypogonadism persisted in 48.2% of men with lower T concentrations over a six-month follow-up post-recovery (66). Even after 12 months of recovery, almost 30% of men still had serum T levels consistent with biochemical hypogonadism. Of clinical relevance, the lower the serum T at admission, the poorer the outcomes and the lower the probability of achieving a state of eugonadal, even after a long period of follow-up (67). Summary reported studies about the impact of COVID-19 on male reproductive hormones are summarized in **Table 3**.

**Table 3:** COVID-19 effect on male reproductive hormones.

Author/year	Number of COVID-19 cases	Main conclusion
Ma et al., 2020 (63)	81	Levels of LH and T were decreased
Çayan et al., 2020 (68)	221	T level decreased
Okçelik, 2021 (69)	44	T level decreased
Lanser et al., 2021 (70)	377	Decreased T levels are associated with increased immunological activation
Kadihasanoglu et al., 2021 (71)	89	Increased LH and prolactin, and decreased tT level
Salonia et al., 2021 (72)	286	Lower T level was related to severe clinical outcomes
Schroeder et al., 2021 (73)	50	Lower T levels may be related to disease severity
Apaydin et al., 2022 (66)	81	Lower T level at correlated with higher inflammatory marker levels
Cinislioglu et al., 2022 (74)	358	Lower tT level indicate worse prognosis.
Salonia et al., 2023 (67)	121 at 7 months FU 63 at 12 months FU	50% and 30% had hypogonadism at 7- and 12-months FU, respectively.

T, testosterone; LH, Luteinizing hormone; tT, Total testosterone; FU, Follow up.

Furthermore, autopsy analysis from the testicular tissue of patients with COVID-19 found that Leydig cells were significantly reduced in the testicular interstitium, which suggests that SARS-CoV-2 may have caused ultrastructural damage to the cells. ACE2 was diffusely expressed in Sertoli cells and strongly expressed in Leydig cells, as revealed by immunostaining (15).

## 4 COVID-19 vaccine’s effect on male reproductive and sexual health

While there has been evidence of the effect of SARS-CoV-2 on sperm parameters, studying the impact of the COVID-19 vaccine is of equal importance, if not more. Not only assuring the vaccine’s safety but also addressing the fears of the general population and increasing acceptance of the vaccine. Several prospective cohort studies have assessed the effect of the SARS-CoV-2 vaccine on semen parameters and found evidence for changes in semen parameters (75, 76). However, one cohort study found that there has been an increase in sperm concentration (77), and another case report found that patients with ankylosing spondylitis who were vaccinated had improved morphology compared to those who were unvaccinated (78). Another study that tested the effect of both the mRNA vaccine and the viral vector vaccine showed no impact on sperm quality (79). Similar results were replicated by multiple studies (79–85). Summary of the reported studies about the impact of COVID-19 vaccine on semen parameters are summarized in **Table 4**.

**Table 4:** Effects of COVID-19 vaccine on semen parameters.

Author/year	Number of sperm donors vaccinated with Covid-19 vaccine	Main conclusion
Alenzi et al., 2022 (82)	100	Isolated increase in progressive sperm motility within physiological limits
Barda et al., 2022 (77)	33	No effect on semen parameters
Chatzimeletiou et al., 2022 (78)	?	Isolated increase in sperm concentration
Gonzalez et al., 2021 (80)	45	No effect on semen parameters
Lifshitz et al., 2022 (83)	75	No effect on semen parameters
Reschini et al., 2022 (79)	106	No effect on semen parameters
Safrai et al., 2022 (85)	72	No effect on semen parameters

Since a meta-analysis was published that showed no significant effect of the mRNA vaccine on semen parameters (86). And following that, a more considerable multinational analysis found no association between BNT162b2 and mRNA-1273 and sub-fertility in men (87).

Other factors studied in correlation to the COVID-19 vaccine include male reproductive hormones. A study conducted by Adamyan et al. found no effect of the SARS-CoV-2 vaccine on the level of testosterone, FSH, LH, or Estradiol hormones (88). Another study showed that the mRNA vaccine showed no association with the risk of developing orchitis or epididymitis, as reported for the SARS-CoV-2 infection (89). Furthermore, when the rate of orchitis and epididymitis was compared between vaccinated and non-vaccinated participants, the rate was significantly lower in vaccinated participants after only a single dose (90). Several systematic reviews and other reviews conclude to find no significant negative effect of the SARS-CoV-2 vaccine on male reproductive health (88, 91). As for Sexual Health, in a large study that isolated all reported urological symptoms from a sample of 15,785 participants, no symptoms related to Erectile function, ejaculatory function, or sexual function were reported (92). Furthermore, a prospective questionnaire-based study concluded that the COVID-19 vaccine did not affect male sexual function (93).

## 5 Limitation

This review is not without limitations. The study concentrated on papers published within a defined time frame and in specific databases, which may have eliminated relevant articles. Nonetheless, the review provided valuable updated insights into the impact of the COVID-19 pandemic on men’s sexual and reproductive health.

## 6 Recommendations and future direction

COVID-19 and its control measures appear to disproportionately impact men’s and women’s sexual and reproductive outcomes. As a result of the COVID-19 pandemic response, the availability of sexual and reproductive services, as well as access to family planning and contraception, has significantly decreased. Men infected with COVID-19 have more severe illness and a greater fatality rate than women. Understanding why males are more prone than women to suffer serious diseases can aid in developing effective therapies, public health policies, and focused tactics such as early detection and intensive testing in subgroups.

## 7 Conclusion

Our review attempted to synthesize the published literature about the impact of the COVID-19 pandemic on sexual and reproductive health among men. To date, many studies reported controversial data specifically related to the COVID-19 pathophysiology on men’s sexual and reproductive experiences during the pandemic, which warrants further clinical investigation.

## Author contributions

MA, RM, and SB participated in the design of this study. MA and RM conducted the literature search. MA, RM, and SB retrieved and selected the articles. MAA and KA conducted the data extraction. MA, KA, AA, and JA wrote the manuscript draft. MA supervised the study. All authors listed have made a substantial, direct, and intellectual contribution to the work and approved it for publication.
